# Association between the serum uric acid-to-creatinine ratio index and the risk of preeclampsia in advanced maternal age pregnant women: a retrospective cohort study

**DOI:** 10.3389/fcvm.2026.1749915

**Published:** 2026-04-02

**Authors:** Ting Zhang, Li Liang, Sulan Huang

**Affiliations:** Department of Cardiology, Changde Hospital, Xiangya School of Medicine, Central South University (The First people’s Hospital of Changde City), Changde, China

**Keywords:** advanced maternal age, preeclampsia, retrospective cohort study, serum uric acid-to-creatinine ratio, SUA/sCr

## Abstract

**Background:**

Advanced maternal age is associated with an increased risk of preeclampsia;however, reliable and easily accessible biomarkers for early risk stratiﬁcation remain limited. The serum uric acid–to–creatinine ratio (SUA/sCr) has been proposed as an indicator of systemic oxidative stress, yet evidence regarding its association with preeclampsia remains limited. This study aimed to examine the association between SUA/sCr levels and the risk of preeclampsia among women of advanced maternal age.

**Methods:**

a total of 2,296 pregnant women aged ≥35 years were included in this retrospective cohort study. Multivariable logistic regression models were applied to evaluate the association between SUA/sCr and preeclampsia, with odds ratios (ORs) and 95% conﬁdence intervals (CIs) reported. Restricted cubic spline regression was used to assess potential dose-response and non-linear relationships, and subgroup analyses were conducted to explore potential effect modiﬁcation.

**Results:**

Preeclampsia occurred in 14.29% of the participants. After multivariable adjustment,each standard deviation increase in SUA/sCr was associated with a higher risk of preeclampsia(OR = 1.29; 95% CI: 1.13–1.47; *P* = 0.0001). Restricted cubic splines suggested no strong evidence of nonlinearity (*P* = 0.354), while a two-piecewise model indicated a suggestive change-point, with a suggested inflection point at an SUA/sCr value of approximately 8.18 (95% CI: 7.60–8.55);However, the threshold effect did not reach conventional statistical significance. Below this value, SUA/sCr was positively associated with preeclampsia risk, whereas no significant association was observed above it. The calculated E-value (1.73) indicated moderate robustness to unmeasured confounding. In subgroup analyses, the association differed by proteinuria status (*P* for interaction = 0.0146), which should be interpreted cautiously and warrants external validation.

**Conclusion:**

Higher SUA/sCr levels in early pregnancy were associated with an increased risk of preeclampsia among women of advanced maternal age. These findings suggest that SUA/sCr may serve as a potential risk indicator in this population; however, the observed non-linear relationship should be interpreted cautiously and warrants further prospective validation.

## Introduction

Preeclampsia is a major subtype of hypertensive disorders of pregnancy (HDP) and remains a leading contributor to maternal and perinatal mortality worldwide (1–3). It is a multisystem condition arising from aberrant placental development at the maternal–fetal interface, ultimately leading to widespread endothelial dysfunction and organ injury (4). Clinically, preeclampsia is diagnosed by new-onset hypertension accompanied by proteinuria or other signs of end-organ damage after 20 weeks of gestation (5). Gestational hypertension is deﬁned as elevated blood pressure (systolic ≥140 mmHg and/or diastolic ≥90 mmHg) on two separate measurements at least 4 h apart, or a single reading of systolic ≥160 mmHg or diastolic ≥110 mmHg in previously normotensive women (3). Proteinuria is typically deﬁned as ≥300 mg per 24 h or a protein-to-creatinine ratio ≥0.3 mg/dL (3, 5).The hypertensive phenotype of preeclampsia reﬂects increased systemic vascular resistance,heightened afterload, and reduced intravascular volume and cardiac output (6). Despite decades of investigation, the underlying mechanisms of preeclampsia remain incompletely understood,and its prevalence continues to rise. The disorder affects approximately 2%–8% of pregnancies and represents a persistent global health challenge (7).Women of advanced maternal age, conventionally deﬁned as ≥35 years at delivery, constitute a population at increased risk for preeclampsia. With ongoing demographic shifts and delayed childbearing worldwide, the burden of hypertensive complications among women of advanced maternal age has become increasingly prominent. Compared with younger pregnant women, this group often exhibits a higher prevalence of baseline vascular dysfunction and metabolic abnormalities, which may predispose them to abnormal placentation and endothelial injury during pregnancy. However, effective early biomarkers speciﬁcally evaluated in women of advanced maternal age remain limited.

Serum uric acid is the ﬁnal metabolite of purine degradation. More than a century ago, Slemons et al. ﬁrst observed markedly elevated uric acid levels in women with eclampsia (8), and since then hyperuricemia has been repeatedly implicated as a clinical marker and potential contributor to preeclampsia (9). Increasing evidence suggests that uric acid participates in several pathological pathways relevant to hypertension and renal injury (9, 10), many of which overlap with the characteristic features of preeclampsia. Experimental studies have demonstrated that elevated uric acid levels can provoke endothelial dysfunction, oxidative stress, and mitochondrial impairment (11). In the context of preeclampsia, uric acid may exacerbate vascular endothelial injury, disrupt spiral artery remodeling, and contribute to shallow trophoblast invasion, ultimately resulting in placental malperfusion and downstream hypoxia affecting both the placenta and the fetus (12). These processes are key drivers in the progression of maternal disease and in fetal complications such as intrauterine growth restriction. Because hyperuricemia may arise from impaired renal urate excretion or excessive xanthine oxidase–mediated production, the serum uric acid/creatinine ratio (SUA/sCr) has emerged as a potentially more informative marker than uric acid alone, as it partially adjusts for renal function and better reﬂects endogenous uric acid load (13). Increasing data suggest that hypertensive disorders of pregnancy—particularly preeclampsia—represent maternal vascular disorders characterized by endothelial dysfunction, impaired angiogenesis, and increased long-term cardiovascular risk (14, 15). Hyperuricemia secondary to increased xanthine oxidase activity can generate reactive oxygen species and has been strongly linked to endothelial and vascular injury (16), mechanisms believed to contribute to the onset, progression, and severity of preeclampsia (17). Despite growing interest in maternal biomarkers, research on the uric acid-to-creatinine ratio(SUA/sCr) in relation to preeclampsia remains limited. A recent study by Piani et al. reported that SUA/sCr may help predict preeclampsia and its associated complications (18).

However, existing evidence has not speciﬁcally addressed its predictive value in women of advanced maternal age, a group with a higher baseline risk of preeclampsia and distinct vascular characteristics.Therefore, the present study aimed to determine whether the SUA/sCr ratio could serve as a reliable predictor of preeclampsia among pregnant women aged over 35 years.

## Methods

### Study population

This study included pregnant women of advanced maternal age (≥35 years) who attended their first-trimester booking visit at the antenatal clinic of the Department of Obstetrics, Changde Hospital, China, between January 2013 and December 2023. At enrollment, the median gestational age was 12.0 weeks (IQR, 10.1–13.8). Data were derived from an ongoing, nonselective registry and were de-identified prior to analysis. Maternal characteristics were collected using semi-structured questionnaires administered by trained interviewers who received standardized instruction on study objectives, procedures, confidentiality, and informed consent; data collection was monitored to ensure protocol adherence and quality control. Written informed consent was obtained from all participants, and the study protocol was approved by the institutional ethics committee.To establish the temporal relationship between SUA/sCr and subsequent preeclampsia, serum uric acid (SUA) and serum creatinine (sCr) were measured at the initial antenatal visit, before any clinical diagnosis of preeclampsia. The absence of post-diagnosis SUA/sCr measurements was verified by cross-checking laboratory test dates against diagnosis dates.

**Figure 1:**
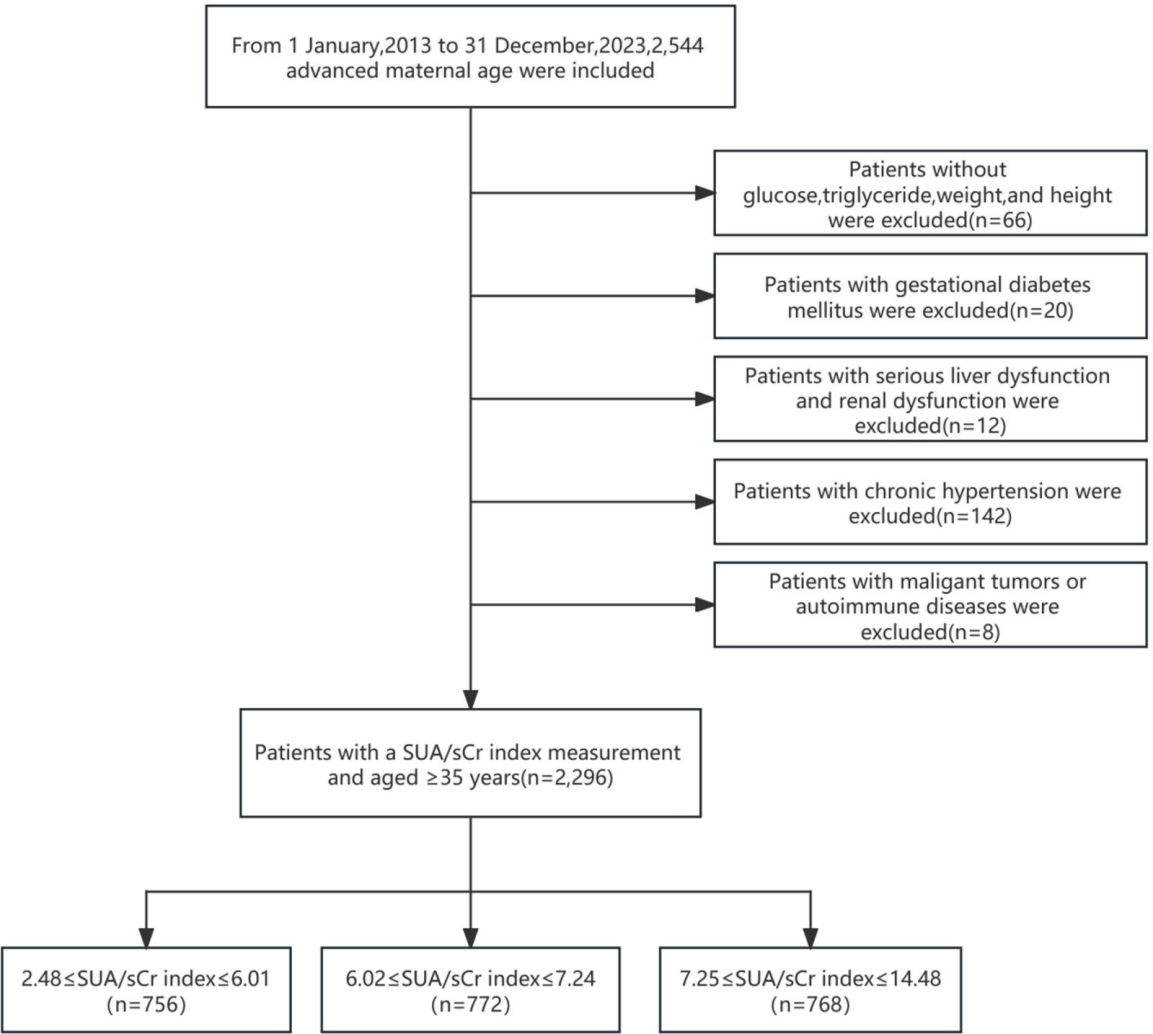
Flow diagram of the screening and enrolment of study patients.

A total of 2,544 naturally conceived pregnancies in women of advanced maternal age were initially screened.The inclusion criteria were maternal age ≥35 years, singleton gestation, and available baseline data required for analysis. Exclusion criteria included missing post-admission measurements of blood glucose, triglycerides, body weight, or height (*n* = 66); gestational diabetes mellitus (*n* = 20); severe hepatic or renal dysfunction (*n* = 12); malignancy or autoimmune disease (*n* = 8); and pre-existing chronic hypertension (*n* = 142). After applying these criteria, 2,296 pregnancies were eligible for analysis, comprising 328 women with preeclampsia and 1,968 without preeclampsia (Figure 1).

### Calculation and categorization of SUA/sCr index

The SUA/sCr index is calculated as the serum uric acid (μmol/L) divided by creatinine (μmol/L), resulting in a dimensionless ratio (19). The SUA/sCr index of the study participants was categorized into three tertiles based on their baseline values: Tertile 1 (T1): 5.17 (4.49–5.70), Tertile 2 (T2): 6.62 (6.31–6.94), and Tertile 3 (T3): 8.30 (7.73–8.93).

### Laboratory analysis

Various maternal characteristics were collected from the hospital's electronic medical record system, including maternal age, body mass index, gestational age, mode of delivery, parity, pregnancy status, and pregnancy complications. All variables were collected during early pregnancy. Maternal blood glucose, lipid levels, creatinine, blood urea nitrogen (BUN), and serum uric acid were measured using hospital laboratory information system data. Blood tests were conducted in the morning after an 8 to 10 h fast. Plasma glucose was measured using the glucose oxidase method. Total cholesterol (TC) and triglycerides (TG) were determined via the enzyme colorimetric method. High-density lipoprotein cholesterol (HDL-c) and low-density lipoprotein cholesterol (LDL-c) were measured using the direct enzymatic method. Consequently, all laboratory analyses were performed by laboratory personnel unaware of gestational age. Follow-up continued until delivery or the occurrence of preeclampsia (PE). Outcomes were ascertained from diagnoses recorded in the outpatient and inpatient electronic medical records.

### Statistical analysis

To ensure the robustness of the statistical methods used in this study, we consulted a statistical expert for an independent evaluation of the analyses, particularly regarding the handling of missing data.Missing data were handled as follows: records with >10% missing values across study variables were excluded, and remaining records (≤10% missing) were imputed using multiple imputation by chained equations (MICE) under the missing-at-random assumption. Twenty imputed datasets were generated (10 iterations each). Continuous variables were imputed using predictive mean matching, and binary/categorical variables were imputed using logistic or multinomial logistic regression, as appropriate. Each imputed dataset was analyzed separately, and effect estimates and standard errors were pooled using Rubin's rules (20).

Continuous variables with a normal distribution were summarized as mean ± standard deviation, whereas those with a non-normal distribution were presented as median (interquartile range). Categorical variables were reported as counts and percentages. For comparisons across the three groups, one-way analysis of variance (ANOVA) was applied to normally distributed variables with homogenous variances, while the Kruskal–Wallis test was used for variables that did not meet these assumptions. Categorical variables were compared using the chi-square test.To evaluate the association between the SUA/sCr index and the risk of preeclampsia in advanced maternal age, logistic regression models were constructed to estimate odds ratios (ORs) and 95% confidence intervals (CIs). In Model II, we adjusted for several potential confounders, including age; gravidity; parity; family history of hypertension; alanine aminotransferase(ALT);albumin(ALB);body mass index(BMI); white blood cell(WBC); red blood cell(RBC).These variables are pre-defined (*a priori*) rather than obtained through data-driven stepwise regression.The selection of these variables was based on existing literature and our clinical experience. For example, ALT and albumin are closely related to liver function and nutritional status, which may affect serum uric acid levels. Additionally, BMI and WBC count are factors related to metabolic stress and inflammation, which are known to be associated with the development of preeclampsia. Therefore, including these variables as adjustment factors helps reduce potential confounding effects and ensures a more accurate assessment of the relationship between SUA/sCr and preeclampsia.

E-values were calculated for statistically significant primary outcomes to assess the potential influence of unmeasured confounding. Likelihood ratio tests were used to examine effect modification and interactions across subgroups. Restricted cubic spline models were employed to characterize the dose-response relationship between the SUA/sCr index and preeclampsia. Smooth curve fitting was further applied to explore potential threshold effects, and piecewise (segmented) regression models were developed when non-linearity was suggested. The optimal inflection points were identified based on the model with the maximum likelihood, implemented using R version 4.4.3 and the mgcv package.

All analyses were performed using SPSS 26.0 (IBM Corp., Armonk, NY, USA), R (http://www.R-project.org), and EmpowerStats (https://www.empowerstats.com, X&Y Solutions, Inc., Boston, MA, USA). A two-sided *p*-value < 0.05 was considered statistically significant.

## Results

### Study population and clinical characteristics results

The baseline characteristics of the study population stratified by the SUA/sCr tertiles are presented in Table 1. A total of 2,296 women of advanced maternal age were included in the analysis. According to baseline SUA/sCr values, 756, 772, and 768 participants were classified into tertile 1 (T1), tertile 2 (T2), and tertile 3 (T3), respectively. Significant differences were observed across SUA/sCr tertiles with respect to several clinical and laboratory parameters. BMI, SUA/sCr index, white blood cell (WBC) count,platelet (PLT) count, red blood cell distribution width (RDW), triglycerides (TG), high-density lipoprotein cholesterol (HDL-c), low-density lipoprotein cholesterol (LDL-c), alanine aminotransferase (ALT), and aspartate aminotransferase (AST) differed significantly among the three groups (all *P* < 0.05). In addition, statistically significant differences were observed in albumin levels, blood urea nitrogen (BUN), fibrinogen concentration, and neutrophil count across tertiles (*P* < 0.05).Regarding demographic and obstetric characteristics, the distribution of maternal age, parity, family history of hypertension, and preeclampsia (PE) differed significantly among the three SUA/sCr groups (*P* < 0.05).The overall incidence of preeclampsia in the study population was 14.29% (328/2,296). Across increasing SUA/sCr tertiles, the proportion of women with preeclampsia increased progressively, with rates of 10.05% in T1, 16.06% in T2, and 16.67% in T3 (*P* < 0.001). This pattern indicates a positive association between higher SUA/sCr levels and the risk of preeclampsia, with the most pronounced increase observed between the first and second tertiles.Compared with women in the lowest tertile (T1), those in the highest tertile (T3) generally exhibited higher BMI, TG, WBC count, RDW, ALT, AST, and fibrinogen levels, as well as lower HDL-c concentrations. In contrast, no statistically significant differences were observed among the three tertiles with respect to gravidity, red blood cell count, hemoglobin, lymphocyte count, potassium levels, total cholesterol (TC), erythrocyte sedimentation rate (ESR), or urine protein (*P* > 0.05).

**Table 1:** Baseline characteristics according to SUA/sCr index tertiles.

SUA/sCr index tertile	Total(*N* = 2,296)	Tertile 1(*N* = 756)	Tertile 2(*N* = 772)	Tertile 3(*N* = 768)	*P*-value
Age(years)	37.00 (36.00–39.00)	38.00 (36.00–40.00)	37.00 (35.00–39.00)	36.00 (35.00–39.00)	<0.001
BMI, kg/m^2^	27.51 (25.28–29.90)	26.44 (24.44–28.84)	27.89 (25.39–29.90)	28.13 (25.89–30.41)	<0.001
Gravidity, num	3.00 (2.00–5.00)	4.00 (3.00–5.00)	3.00 (3.00–5.00)	3.00 (2.00–5.00)	0.101
Parity					<0.001
Nulliparous	472 (20.56%）	132 (17.46%)	192 (24.87%)	148 (19.27%)	
Parous	1,824 (79.44%）	624 (82.54%)	580 (75.13%)	620 (80.73%)	
SUA/sCr index	6.62 (5.71–7.73)	5.17 (4.49–5.70)	6.62 (6.31–6.94)	8.30 (7.73–8.93)	<0.001
WBC, 10^9^/L	8.87 (7.23–10.81)	8.55 (7.26–10.17)	8.87 (6.83–10.41)	8.99 (7.37–11.37)	<0.001
RBC, 10^12^/L	3.75 (3.50–4.03)	3.75 (3.50–4.06)	3.83 (3.52–4.04)	3.75 (3.48–4.01)	0.237
Hemoglobin, g/L	114.00 (106.00–124.00)	116.00 (110.00–124.00)	114.00 (107.00–125.00)	113.00 (105.75–125.00)	0.054
RDW, %	14.00 (13.30–14.90)	13.80 (13.20–14.40)	14.20 (13.30–15.10)	14.10 (13.40–14.93)	<0.001
PLT, 10^9^/L	186.00 (153.00–220.00)	188.00 (163.00–214.00)	191.00 (155.00–224.00)	172.00 (144.00–221.75)	<0.001
Lymphocyte, 10^9^/L	1.11 (0.71–1.65)	1.10 (0.71–1.61)	1.17 (0.71–1.71)	1.07 (0.67–1.59)	0.064
Neutrophil, 10^9^/L	5.45 (3.95–10.82)	4.99 (3.87–10.61)	5.76 (4.03–10.99)	5.83 (3.95–11.02)	0.036
Glucose, mmol/L	4.92 (4.24–5.73)	4.93 (4.36–5.79)	4.69 (4.15–5.61)	4.99 (4.14–5.81)	0.005
TG, mmol/L	3.99 ± 1.46	3.72 ± 0.68	3.93 ± 0.87	4.33 ± 2.23	<0.001
TC, mmol/L	6.13 ± 0.86	6.11 ± 0.59	6.09 ± 0.85	6.19 ± 1.06	0.706
HDL-c, mmol/L	1.84 ± 0.33	1.85 ± 0.21	1.81 ± 0.25	1.87 ± 0.46	<0.001
LDL-c,mmol/L	3.06 ± 0.54	3.06 ± 0.34	3.08 ± 0.58	3.04 ± 0.65	0.002
ALT, U/L	13.50 (10.00–21.00)	13.00 (10.00–18.00)	15.00 (10.00–22.00)	14.00 (10.00–25.00)	<0.001
AST, U/L	20.00 (17.00–26.00)	19.00 (17.00–24.00)	19.00 (16.00–27.00)	21.00 (17.00–27.00)	<0.001
Albumin, g/L	35.30 (33.70–37.40)	35.20 (33.50–37.70)	35.00 (33.10–37.00)	35.80 (33.98–37.85)	<0.001
BUN, mmol/L	3.28 (2.76–4.04)	3.34 (2.67–4.22)	3.20 (2.74–3.89)	3.29 (2.83–3.97)	0.014
Potassium, mmol/L	3.93 (3.77–4.10)	3.93 (3.77–4.10)	3.95 (3.77–4.14)	3.93 (3.74–4.08)	0.239
Fibrinogen, g/L	4.37 (3.81–4.97)	4.26 (3.63–4.97)	4.37 (3.81–4.85)	4.46 (3.86–5.11)	<0.001
ESR, mm/h	37.62 ± 7.93	37.51 ± 4.52	37.74 ± 9.33	37.62 ± 8.96	0.314
Family history of hypertension					0.004
No	1,671 (72.78%)	583 (77.12%)	540 (69.95%)	548 (71.35%)	
Yes	625 (27.22%)	173 (22.88%)	232 (30.05%)	220 (28.65%)	
PE					<0.001
No	1,968 (85.71%)	680 (89.95%)	648 (83.94%)	640 (83.33%)	
Yes	328 (14.29%)	76 (10.05%)	124 (16.06%)	128 (16.67%)	
Urine protein					0.140
No	1,256 (54.70%)	424 (56.08%)	400 (51.81%)	432 (56.25%)	
Yes	1,040 (45.30%)	332 (43.92%)	372 (48.19%)	336 (43.75%)	

Data are presented as means ± SDs, medians (interquartile ranges), or *n* (%). BMI, body mass index; RDW, red cell distribution width; WBC, white blood cell; RBC, red blood cell; PLT, Platelet; ALT, alanine aminotransferase; AST, aspartate aminotransferase; BUN, blood urea nitrogen; TG, Triglyceride; TC, total cholesterol; HDL-c, high density lipoprotein cholesterol; LDL-c, low density lipoprotein cholesterol; PE, preeclampsia; ESR, Erythrocyte sedimentation rate; SUA/sCr, Uric acid/creatinine ratio;.

### Univariate and multivariate logistic analysis results

In univariate analysis treating the SUA/sCr index as a continuous variable, higher SUA/sCr was significantly associated with increased odds of preeclampsia (OR = 1.15; 95% CI: 1.08–1.22; *P* < 0.0001) (Supplementary Table S1) (Table 2). This positive association remained statistically significant after adjustment for potential confounders in both multivariable models: Model I (OR = 1.15; 95% CI: 1.08–1.23; *P* < 0.0001) and Model II (OR = 1.16; 95% CI: 1.07–1.24; *P* = 0.0001).When the SUA/sCr index was expressed per standard deviation (SD) increase, the risk of preeclampsia increased by approximately 27%–29% across all models. Specifically, the unadjusted model yielded OR = 1.27 (95% CI: 1.14–1.42; *P* < 0.0001), Model I yielded OR = 1.28 (95% CI:1.15–1.44; *P* < 0.0001), and Model II yielded OR = 1.29 (95% CI: 1.13–1.47; *P* = 0.0001).When participants were categorized into tertiles, women in the highest SUA/sCr tertile (T3) had a significantly higher risk of preeclampsia compared with those in the lowest tertile (T1). In the crude analysis, the OR for T3 vs. T1 was 1.79 (95% CI: 1.32–2.42; *P* = 0.0002). In Model I, the OR was 1.76 (95% CI: 1.30–2.39; *P* = 0.0003). After further adjustment in Model II, the association remained statistically significant, with OR = 1.48 (95% CI: 1.06–2.07; *P* = 0.0227). In contrast, the association for the middle tertile (T2) was attenuated and no longer statistically significant in Model II (*P* = 0.1948).A consistent dose-response relationship across SUA/sCr tertiles was supported by significant *P* for trend values in all models: unadjusted *P* = 0.0002; Model I *P* = 0.0004; Model II *P* = 0.0226.Taken together, these findings indicate that a higher SUA/sCr index is independently associated with an increased risk of preeclampsia among women of advanced maternal age.

**Table 2:** The association between SUA/sCr index and preeclampsia (*n* = 328) by multivariate logistic regression analysis.

Exposure	Non-adjusted model		Adjust I model		Adjust II model	
	OR(95%CI)	*P*	OR(95%CI)	*P*	OR(95%CI)	*P*
Continuous	1.15 (1.08, 1.22)	<0.0001	1.15 (1.08, 1.23)	<0.0001	1.16 (1.07, 1.24)	0.0001
Per SD increase	1.27 (1.14, 1.42)	<0.0001	1.28 (1.15, 1.44)	<0.0001	1.29 (1.13, 1.47)	0.0001
Tertile of SUA/sCr						
Tertile1	1.0		1.0		1.0	
Tertile2	1.71 (1.26, 2.32)	0.0006	1.68 (1.24, 2.28)	0.0009	1.24 (0.89, 1.72)	0.1948
Tertile3	1.79 (1.32, 2.42)	0.0002	1.76 (1.30, 2.39)	0.0003	1.48 (1.06, 2.07)	0.0227
*P* for trend	1.31 (1.13, 1.52)	0.0002	1.30 (1.13, 1.51)	0.0004	1.21 (1.03, 1.43)	0.0226

Non-adjusted model adjust for: None. Adjust I model adjust for: age; gravidity; parity. Adjust II model adjust for: age; gravidity; parity; family history of hypertension; ALT; ALB; BMI; WBC; RBC. CI, confidence interval; OR, odds ratio.

### Value analysis results

**Figure 2:**
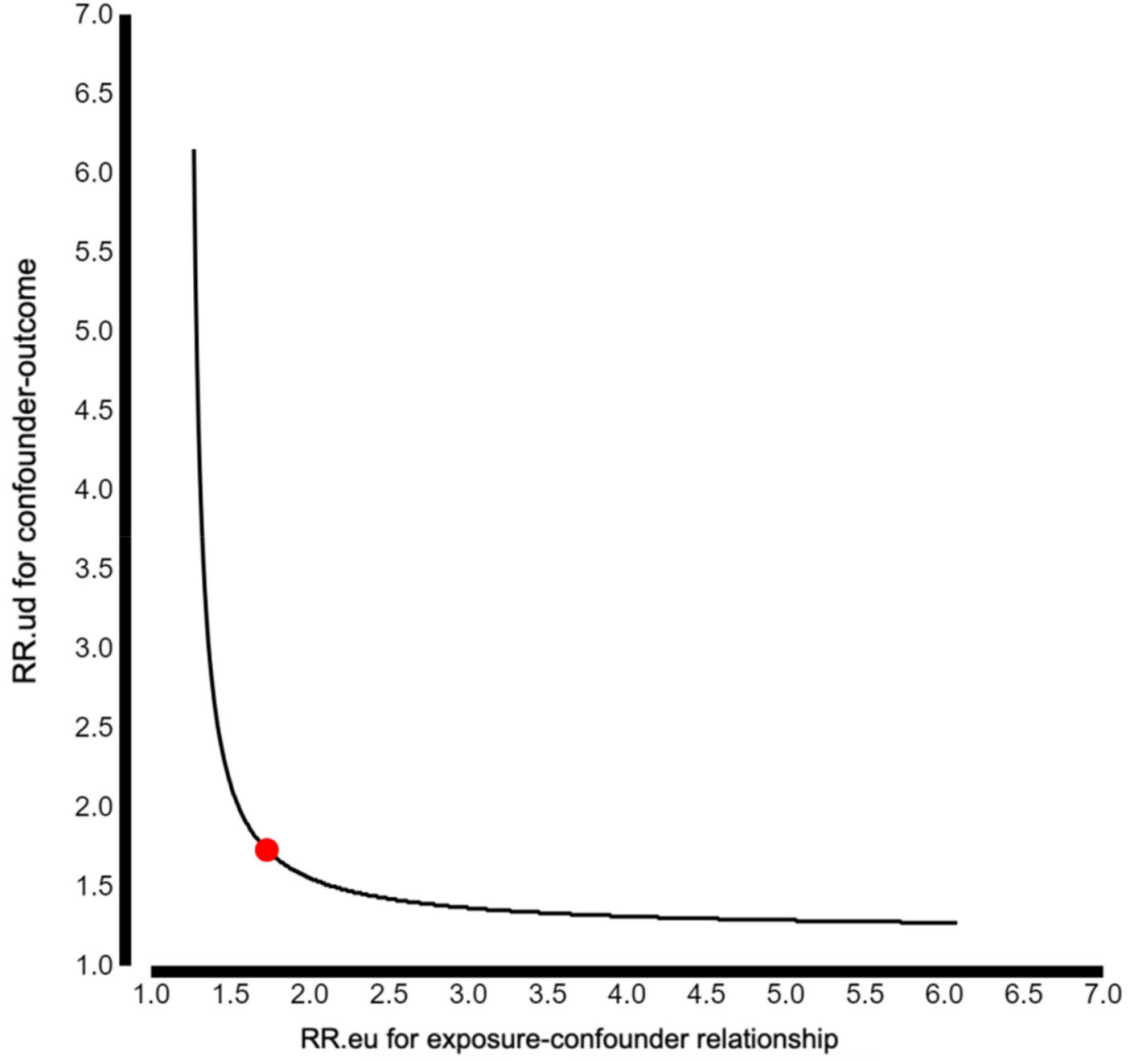
E-value analyses.

Using the E-value approach proposed by VanderWeele and Ding (21), a sensitivity analysis was conducted to assess the potential impact of unmeasured confounding on the observed association between the SUA/sCr index and preeclampsia. The E-value for the observed point estimate was 1.73, and the corresponding E-value for the lower bound of the 95% conﬁdence interval was 1.46.These values indicate that, to fully explain away the observed association, an unmeasured confounder would need to be associated with both the SUA/sCr index and preeclampsia by a risk ratio of at least 1.73,independent of the measured covariates.Alternatively,an unmeasured confounder associated with both exposure and outcome by a risk ratio of 1.46 would be sufficient to shift the lower conﬁdence limit to the null.As illustrated in Figure 2, assuming a moderate association between a hypothetical unmeasured confounder and the SUA/sCr index (RR_eu = 1.5), the strength of association between the confounder and preeclampsia (RR_ud) would need to reach approximately 2.3 to negate the observed effect estimate.Such a combination of confounder–exposure and confounder–outcome associations would represent a relatively strong confounding structure in the context of clinical epidemiological research.Overall, the E-value analysis suggests that the observed association between the SUA/sCr index and preeclampsia is moderately robust to potential unmeasured confounding,although residual confounding cannot be entirely excluded.

### Analysis of the association between the SUA/sCr index and the incidence of preeclampsia

**Figure 3:**
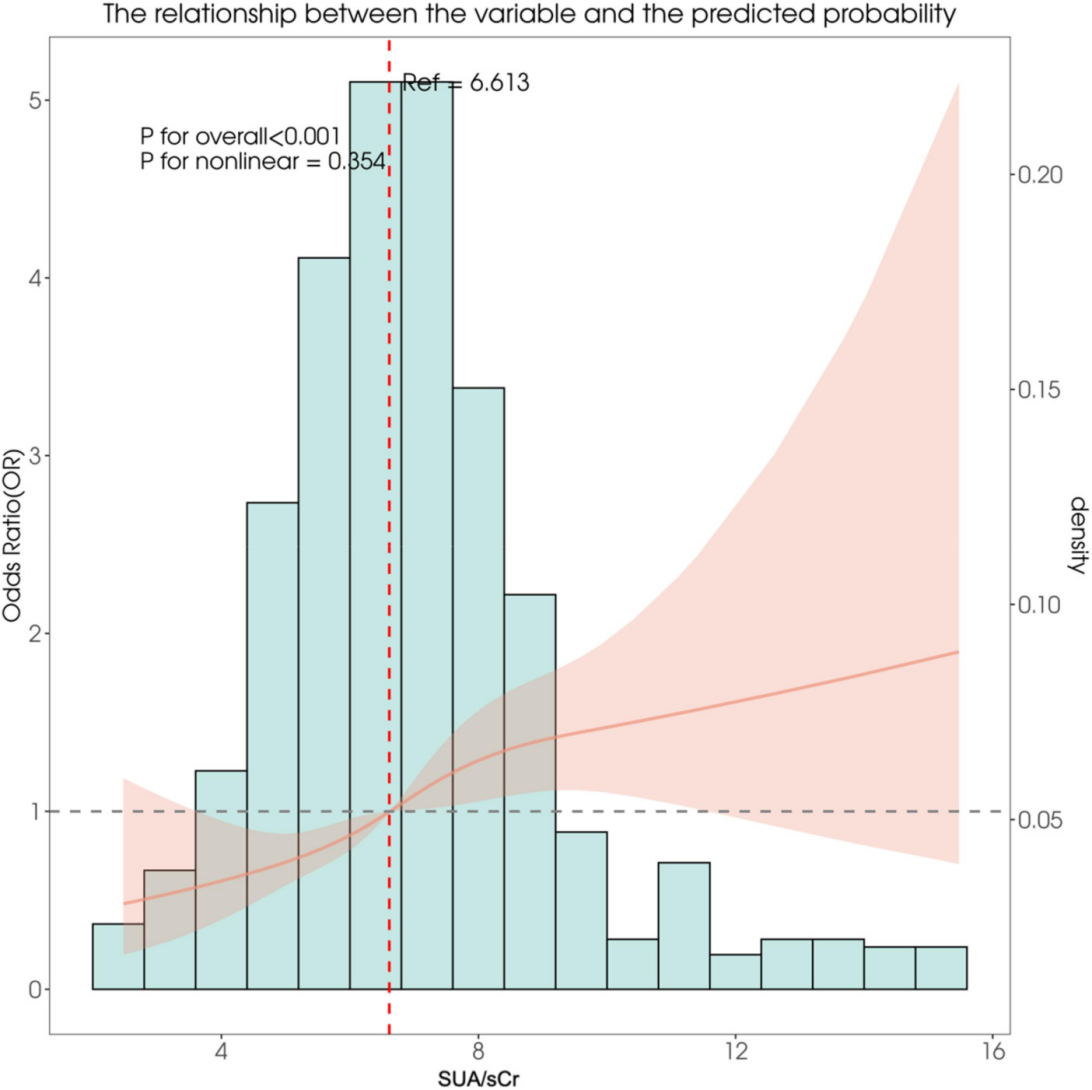
Restricted cubic spline curves for preeclampsia by SUA/sCr index.

As shown in Figure 3, the potential non-linear association between the SUA/sCr index and the risk of preeclampsia was explored using restricted cubic spline models after adjustment for age,gravidity, and parity. The overall association between the SUA/sCr index and preeclampsia was statistically significant (*P* for overall < 0.001), while the test for nonlinearity did not reach statistical significance (*P* for nonlinearity = 0.354), suggesting that the relationship was not strongly nonlinear across the full exposure range.To further investigate possible threshold effects, a two-piecewise linear regression model was applied (Table 3). The inﬂection point (K) of the SUA/sCr index a suggestive change-point around 8.18 (95% CI: 7.60–8.55). When the SUA/sCr index was below this threshold (<8.18), a significant positive association with preeclampsia risk was observed (OR = 1.23; 95% CI: 1.11–1.36; *P* < 0.0001). In contrast, when the SUA/sCr index was at or above the threshold (≥8.18), the association was no longer statistically significant (OR = 1.02; 95% CI: 0.89–1.18; *P* = 0.7561).The likelihood-ratio test comparing the piecewise model with a single linear model yielded a *P* value of 0.068, indicating a suggestive but not statistically significant improvement in model ﬁt with the inclusion of a threshold term. Collectively, these findings suggest that the risk of preeclampsia increases predominantly within the lower range of SUA/sCr values, while the association appears to attenuate at higher levels.

**Table 3:** Threshold effect analysis of the relationship between the SUA/sCr index and preeclampsia using a two piece-wise regression model.

SUA/sCr index inflection point	preeclampsia OR(95%CI)	*P*
<8.18	1.23 (1.11, 1.36)	<0.0001
≥8.18	1.02 (0.89, 1.18)	0.7561
Likelihood-ratio test		0.068

The threshold (K) was estimated at 8.18 (95% CI: 7.60–8.55).

CI, confidence interval; OR, odds ratio.

### Stratification analysis on the association between SUA/sCr Index with risk of preeclampsia

The results of the subgroup analyses are summarized in Table 4. After adjustment for potential confounding factors, no statistically significant interactions were observed between the SUA/sCr index and gravidity, parity, family history of hypertension,hemoglobin level, or serum potassium concentration (all interaction *P* > 0.05).In contrast, a statistically significant interaction was identiﬁed for urinary protein status (interaction *P* = 0.0146). In the subgroup without proteinuria, the SUA/sCr index was not significantly associated with the risk of preeclampsia (OR = 1.04; 95% CI: 0.93-1.16; *P* = 0.4848). However, among participants with positive urinary protein, a significant positive association was observed between the SUA/sCr index and preeclampsia risk (OR = 1.23; 95% CI: 1.13-1.34; *P* < 0.001).Overall, the association between the SUA/sCr index and preeclampsia was generally consistent across most subgroups. The magnitude of the association appeared slightly stronger among women with a higher number of pregnancies (≥3) and among those without anemia (*P* < 0.0001), suggesting that the SUA/sCr index may more clearly reflect preeclampsia risk in these populations.

**Table 4:** Stratification analysis on the association between SUA/sCr index and preeclampsia.

Covariates	No	OR(95%CI)	Sub-group *P* value	*P* for interaction
Gravidity, num				0.3757
<3	596	1.08 (0.94, 1.25)	0.2553	
≥3	1,700	1.16 (1.08, 1.25)	<0.0001	
Parity				0.1726
Nulliparous	472	1.02 (0.85, 1.23)	0.8416	
Parous	1,824	1.17 (1.09, 1.25)	<0.0001	
Family history of hypertension				0.1619
No	1,671	1.18 (1.08, 1.09)	<0.0001	
Yes	625	1.08 (0.97, 1.19)	0.1459	
Hemoglobin, g/L				0.5872
<90	88	1.28 (0.87 1.87)	0.2130	
≥90	2,208	1.15 (1.07, 1.22)	<0.0001	
Potassium, mmol/L				0.1417
<3.5	100	2.29 (0.77, 6.82)	0.1369	
≥3.5	2,196	1.14 (1.07, 1.22)	<0.0001	
Urine protein				0.0146
No	1,256	1.04 (0.93, 1.16)	0.4848	
Yes	1,040	1.23 (1.13, 1.34)	<0.001	

### Sensitivity analysis: SUA/sCr and preeclampsia risk in women assessed before 20 gestational weeks without baseline proteinuria (*n* = 1,256)

We conducted a sensitivity analysis restricted to women with SUA/sCr measured before 20 gestational weeks and excluding those with baseline proteinuria (*n* = 1,256). In this restricted subset, SUA/sCr was not significantly associated with preeclampsia in the non-adjusted model (OR = 1.04, 95% CI: 0.93–1.16; *P* = 0.4848) or the minimally adjusted model (Adjust I: OR = 1.05, 95% CI: 0.94–1.17; *P* = 0.4240), but became statistically significant after full adjustment (Adjust II: OR = 1.25, 95% CI: 1.08–1.45; *P* = 0.0031). Findings were consistent when SUA/sCr was analyzed as a *Z*-score (Adjust II: OR = 1.48, 95% CI: 1.14–1.93; *P* = 0.0031). When analyzed by tertiles, neither the middle nor high tertile differed significantly from the lowest tertile in any model (e.g., high vs. low in Adjust II: OR = 1.57, 95% CI: 0.89–2.76; *P* = 0.1176; middle vs. low in Adjust II: OR = 1.00, 95% CI: 0.57–1.76; *P* = 0.9968). Treating tertiles as an ordinal variable showed a non-significant trend in the fully adjusted model (Adjust II: OR = 1.28, 95% CI: 0.96–1.70; *P* = 0.0963), which should be interpreted cautiously given the reduced sample size in this restricted analysis(Shown in Supplementary Table S2).

## Discussion

In this retrospective cohort study of women of advanced maternal age, higher SUA/sCr was associated with increased preeclampsia risk. While piecewise regression suggested an apparent plateau at higher SUA/sCr values (around 8.18), formal model comparison showed that the threshold model did not significantly improve fit over a linear specification (likelihood-ratio test *P* = 0.068), and restricted cubic splines provided no evidence of nonlinearity (*P* for nonlinearity = 0.354); Accordingly, the estimated change-point should be considered exploratory and should not be used as a clinical decision threshold. The plateau-like pattern may also reflect methodological factors rather than a true biological threshold, including fewer observations at the upper range (reduced precision), measurement error/regression dilution, and differential clinical management at higher perceived risk, all of which could attenuate associations at the extremes. Subgroup analyses suggested a potentially stronger association between SUA/sCr and preeclampsia among women with positive urinary protein; however, given the exploratory nature of these analyses and the possibility of residual confounding, this finding should be interpreted with caution and warrants confirmation in future studies.Overall, these findings indicate that SUA/sCr could serve as a marker correlated with early physiological changes during preeclampsia development (e.g., altered maternal hemodynamic adaptation and metabolic stress), although causality cannot be inferred from the present observational design.

### Mechanisms underlying the SUA/sCr Index and preeclampsia

From a physiological perspective, compared with pre-pregnancy levels, serum uric acid and creatinine levels show a downward trend in the first and second trimesters of pregnancy, while they gradually increase in the second and third trimesters (22, 23). This dynamic change is regulated by multiple factors, including the blood dilution effect, increased blood volume and cardiac output, vascular resistance regulation, alterations in glomerular filtration rate and secretory function, fluctuations in hormone levels (such as estrogen, progesterone, and diuretic hormone), decreased albumin levels, and changes in metabolic substrates required for placental and fetal growth (24–26). These changes collectively reflect the significant cardiovascular and hemodynamic adaptations required for normal pregnancy.Preeclampsia is regarded as a syndrome of poor hemodynamic and endothelial function adaptation during pregnancy, characterized by enhanced vasoconstriction, endothelial injury, reduced renal perfusion, and increased systemic inflammation and oxidative stress (27). The ratio of serum uric acid to creatinine (SUA/sCr) is regarded as an indicator of “renal-function-normalized SUA” (28), and existing studies have shown that its level is related to inflammatory markers (such as hs-CRP) and oxidative stress status (29). Therefore, it can serve as an indirect indicator of these pathological conditions.

Elevated uric acid levels in patients with preeclampsia may be the result of multiple pathological processes. Firstly, the constriction of renal blood vessels and the entry of fetal DNA into the maternal circulation can jointly promote the increase of uric acid production, as fetal DNA is converted into uric acid during the degradation process in the liver (30). Secondly, before clinical symptoms appear, the increase in uric acid produced by the placenta itself may also play a key role (31). In the early stage of pregnancy, the placenta and fetal tissues can endogenous produce fructose, and an important by-product of fructose metabolism is uric acid. In cases of persistent hypoxia or placental hypoplasia, the production of endogenous fructose may increase significantly. In addition, a diet high in sugar or fructose consumed by the mother will further promote an increase in uric acid levels (32). Previous studies have pointed out that excessive sugar intake during pregnancy increases the risk of preeclampsia and adverse perinatal outcomes. Meanwhile, a long-term high-fructose diet before pregnancy can lead to hyperuricemia in the early stage of pregnancy, thereby increasing the susceptibility of pregnant women to preeclampsia (33, 34).

### Comparison with previous studies

Growing evidence suggests that hyperuricemia in early pregnancy has pathogenic effects on the placenta, fetus, and maternal health. Yue et al. conducted a retrospective study involving 4,725 singleton pregnant women, measuring their serum uric acid levels before 20 weeks of gestation. They found a positive correlation between serum uric acid concentration and the risk of preeclampsia (35). Yaki Ttiran et al. compared patients with preeclampsia and healthy controls (*n* = 84 and *n* = 86, respectively) and found that the SUA/sCr ratio was significantly higher in preeclampsia patients and was positively correlated with the platelet/lymphocyte ratio in newborns (36). Piani et al. analyzed the medical records of 269 women in a gestational hypertension outpatient clinic and found that the SUA/sCr ratio continued to increase at all stages of pregnancy in women with preeclampsia. After adjusting for covariates, a higher SUA/sCr ratio in the third trimester was associated with an increased risk of preeclampsia [OR = 1.29, 95% CI: 1.15–1.50, *P* = 0.001] (18). While our study also found that each standard deviation increase in the SUA/sCr ratio in early pregnancy (before 20 weeks) was associated with an OR of 1.29, it is important to highlight that Piani et al.'s findings were observed in the third trimester. The different gestational timing of these studies may result in differing implications for risk prediction. Our study suggests that the SUA/sCr ratio in early pregnancy may serve as an early marker for preeclampsia risk, whereas Piani et al.'s study shows that the relationship becomes more apparent in the third trimester. Therefore, while both studies show a similar association between the SUA/sCr ratio and preeclampsia risk, caution is needed when directly comparing the odds ratios, as they reflect associations at different stages of pregnancy.

### Subgroup analysis and clinical implications

Although observational studies have evaluated serum uric acid and the uric acid-to-creatinine ratio (SUA/sCr) in relation to preeclampsia, evidence specifically focused on pregnant women of advanced maternal age (≥35 years) remains limited. In China, the relaxation of the two-child and three-child policies has contributed to increases in late marriage and childbearing, leading to a growing proportion of pregnancies in women aged 35 and older. This group appears to face a higher risk of preeclampsia than younger women (37–39), underscoring the need for simple, practical approaches to early risk stratification and follow-up in this population.Interpretation of uric acid alone can be complicated by hyperuricemia from heterogeneous causes, which may contribute to inconsistent findings across studies. Some evidence suggests that hyperuricemia related to increased xanthine oxidase activity may be associated with preeclampsia and adverse outcomes (40–42), whereas secondary hyperuricemia driven by impaired renal function can confound the association. The SUA/sCr ratio partially accounts for renal function (via creatinine) and may therefore better reflect urate burden than uric acid alone in settings where kidney function influences serum urate levels.

From a clinical and implementation standpoint, SUA and creatinine are routinely available and relatively low-cost tests, making SUA/sCr feasible to consider as an adjunct measure within routine prenatal assessment. Practically, SUA/sCr may be more appropriate as a continuous marker for early risk stratification, with relatively higher levels (e.g., upper tertile or higher quantiles) identifying women who may merit closer surveillance within standard prenatal care (e.g., more frequent blood pressure monitoring, repeat labs when clinically indicated, and comprehensive assessment of established preeclampsia risk factors). Importantly, SUA/sCr is not diagnostic and should not independently guide treatment decisions.

In the subgroup analyses, the direction of association was broadly similar across most subgroups. Although statistically significant associations were observed in some strata (e.g., gravidity ≥3 parous women, and women with hemoglobin ≥90 g/L), the corresponding interaction tests were not significant, suggesting that these between-stratum differences may reflect chance, differences in sample size, or limited precision rather than true heterogeneity of effect. Therefore, apart from urinary protein status, the subgroup findings should be interpreted as exploratory rather than definitive evidence of differential associations.Proteinuria remains central to preeclampsia diagnosis, but it can be influenced by chronic kidney disease, urinary tract infection, and transient conditions. Because SUA/sCr partially accounts for renal function, it may provide complementary information—particularly before overt proteinuria develops. Our subgroup findings suggest the SUA/sCr–preeclampsia association may be more pronounced among women with positive proteinuria, potentially reflecting underlying renal/endothelial dysfunction; however, this observation should be interpreted cautiously and does not indicate that SUA/sCr is superior to or can replace established diagnostic criteria.

Future prospective studies are needed to confirm these findings in well-powered cohorts and to determine incremental clinical utility beyond existing approaches, including evaluation across gestational windows and diverse populations, external validation, and standardized prediction/clinical utility metrics (e.g., AUC, NRI/IDI, calibration, decision-curve analysis), alongside formal cost-effectiveness analyses prior to routine adoption.

### Limitations and future directions

#### Limitations

This study has several limitations. First, given the retrospective observational design, residual confounding cannot be fully eliminated and causal inference is not possible. Second, our inclusion/exclusion criteria may have introduced selection bias and limited external validity because we excluded women with chronic hypertension, and other comorbidities that commonly coexist with advanced maternal age; therefore, the findings are most applicable to women with advanced maternal age who had singleton spontaneous pregnancies and were free of these major comorbidities at baseline. Third, to reduce reverse causality, SUA and sCr were measured at the initial antenatal visit (median 12.0 weeks; IQR: 10.1–13.8 weeks) and no SUA/sCr measurements occurred after a preeclampsia diagnosis; however, subclinical pathophysiological changes may precede clinical diagnosis, so reverse causality cannot be completely excluded. Finally, several important confounders were unavailable or could not be reliably ascertained, including pre-pregnancy BMI, smoking, socioeconomic indicators, diet, physical activity, and some chronic conditions. Medication exposure during pregnancy (notably aspirin prophylaxis, antihypertensive agents, urate-lowering therapy, and other drugs affecting renal function or uric acid metabolism) was not systematically recorded and could not be adjusted for.

#### Future directions

Future work should (1) develop and externally validate prediction models that integrate SUA/sCr with blood pressure, renal markers, and other clinical parameters in women of advanced maternal age; (2) establish prospective cohorts with repeated measurements across gestation to better define temporal patterns; and (3) assess whether modifiable factors or interventions that influence SUA/sCr are associated with reduced preeclampsia risk, with standardized capture and adjustment for key medication exposures (including aspirin prophylaxis and antihypertensive therapy).

## Conclusions

In conclusion, this study provides evidence that the SUA/sCr index is associated with preeclampsia risk among women of advanced maternal age. Although nonlinear analyses suggested an exploratory change-point, the overall evidence did not support a clinically meaningful threshold. Given its simplicity, low cost, and routine availability, SUA/sCr may serve as an adjunct marker for early risk stratification in this population. Prospective studies are needed to confirm these findings and to further elucidate the biological mechanisms linking SUA/sCr to preeclampsia.

## Data Availability

The datasets presented in this study can be found in online repositories. The names of the repository/repositories and accession number(s) can be found in the article/Supplementary Material.

## References

[1] Roberts JM. Preeclampsia epidemiology(ies) and pathophysiology(ies). Best Pract Res Clin Obstet Gynaecol. (2024) 94:102480. 10.1016/j.bpobgyn.2024.102480 38490067

[2] Opichka MA Rappelt MW Gutterman DD Grobe JL McIntosh JJ. Vascular dysfunction in preeclampsia. Cells. (2021) 10(11):3055. 10.3390/cells10113055 34831277 PMC8616535

[3] ACOG Practice Bulletin No. 202: gestational hypertension and preeclampsia. Obstet Gynecol. (2019) 133(1):1. 10.1097/AOG.0000000000003018 30575675

[4] Jim B Karumanchi SA. Preeclampsia: pathogenesis, prevention, and long-term complications. Semin Nephrol. (2017) 37(4):386–97. 10.1016/j.semnephrol.2017.05.011 28711078

[5] Brown MA Magee LA Kenny LC Karumanchi SA McCarthy FP Saito S The hypertensive disorders of pregnancy: iSSHP classification, diagnosis & management recommendations for international practice. Pregnancy Hypertens. (2018) 13:291–310. 10.1016/j.preghy.2018.05.004 29803330

[6] Mustafa R Ahmed S Gupta A Venuto RC. A comprehensive review of hypertension in pregnancy. J Pregnancy. (2012) 2012:105918. 10.1155/2012/105918 22685661 PMC3366228

[7] Magee LA Nicolaides KH von Dadelszen P. Preeclampsia. N Engl J Med. (2022) 386(19):1817–32. 10.1056/NEJMra2109523 35544388

[8] Slemons JM Bogert LJ. The uric acid content of maternal and fetal blood. J Biol Chem. (1917) 32(1):63–9. 10.1016/S0021-9258(18)86658-3

[9] Kang DH Finch J Nakagawa T Karumanchi SA Kanellis J Granger J Uric acid, endothelial dysfunction and pre-eclampsia: searching for a pathogenetic link. J Hypertens. (2004) 22(2):229–35. 10.1097/00004872-200402000-00001 15076175

[10] Nakagawa T Mazzali M Kang DH Sánchez-Lozada LG Herrera-Acosta J Johnson RJ. Uric acid–a uremic toxin? Blood Purif. (2006) 24(1):67–70. 10.1159/000089440 16361844

[11] Sánchez-Lozada LG Lanaspa MA Cristóbal-García M García-Arroyo F Soto V Cruz-Robles D Uric acid-induced endothelial dysfunction is associated with mitochondrial alterations and decreased intracellular ATP concentrations. Nephron Experimental Nephrology. (2013) 121(3-4):e71–8. 10.1159/000345509 PMC3656428 23235493

[12] Bainbridge SA Roberts JM von Versen-Hoynck F Koch J Edmunds L Hubel CA. Uric acid attenuates trophoblast invasion and integration into endothelial cell monolayers. Am J Physiol Cell Physiol. (2009) 297(2):C440–50. 10.1152/ajpcell.00593.2008 19535510 PMC2724097

[13] Borghi C Piani F. Uric acid and estimate of renal function. Let’s stick together. Int J Cardiol. (2020) 310:157–8. 10.1016/j.ijcard.2020.01.046 31996303

[14] Qu H Khalil RA. Vascular mechanisms and molecular targets in hypertensive pregnancy and preeclampsia. Am J Physiol Heart Circ Physiol. (2020) 319(3):H661–81. 10.1152/ajpheart.00202.2020 32762557 PMC7509272

[15] Tomimatsu T Mimura K Matsuzaki S Endo M Kumasawa K Kimura T. Preeclampsia: maternal systemic vascular disorder caused by generalized endothelial dysfunction due to placental antiangiogenic factors. Int J Mol Sci. (2019) 20(17):4246. 10.3390/ijms20174246 31480243 PMC6747625

[16] Landmesser U Spiekermann S Dikalov S Tatge H Wilke R Kohler C Vascular oxidative stress and endothelial dysfunction in patients with chronic heart failure: role of xanthine-oxidase and extracellular superoxide dismutase. Circulation. (2002) 106(24):3073–8. 10.1161/01.CIR.0000041431.57222.AF 12473554

[17] San Juan-Reyes S Gómez-Oliván LM Islas-Flores H Dublán-García O. Oxidative stress in pregnancy complicated by preeclampsia. Arch Biochem Biophys. (2020) 681:108255. 10.1016/j.abb.2020.108255 31904364

[18] Piani F Agnoletti D Baracchi A Scarduelli S Verde C Tossetta G Serum uric acid to creatinine ratio and risk of preeclampsia and adverse pregnancy outcomes. J Hypertens. (2023) 41(8):1333–8. 10.1097/HJH.0000000000003472 37260263 PMC10328517

[19] Liao L Huang W Ma R Hu W Wu H Su M Serum uric acid to creatinine ratio in patients with early-onset post-stroke cognitive impairment: a retrospective cohort study. Front Aging Neurosci. (2025) 17:1580722. 10.3389/fnagi.2025.1580722 40671786 PMC12263954

[20] Segura-Buisan J Leyrat C Gomes M. Addressing missing data in the estimation of time-varying treatments in comparative effectiveness research. Stat Med. (2023) 42(27):5025–5038. 10.1002/sim.9899 37726937 PMC10947135

[21] Ioannidis JPA Tan YJ Blum MR. Limitations and misinterpretations of E-values for sensitivity analyses of observational studies. Ann Intern Med. (2019) 170(2):108–11. 10.7326/M18-2159 30597486

[22] Boyle JA Campbell S Duncan AM Greig WR Buchanan WW. Serum uric acid levels in normal pregnancy with observations on the renal excretion of urate in pregnancy. J Clin Pathol. (1966) 19(5):501–3. 10.1136/jcp.19.5.501 5919366 PMC473361

[23] Amini E Sheikh M Hantoushzadeh S Shariat M Abdollahi A Kashanian M. Maternal hyperuricemia in normotensive singleton pregnancy, a prenatal finding with continuous perinatal and postnatal effects, a prospective cohort study. BMC Pregnancy Childbirth. (2014) 14(1):104. 10.1186/1471-2393-14-104 24636149 PMC3995428

[24] Fischer RL Weisberg LS Hediger ML. Etiology of third-trimester maternal hyperuricemia in nonpreeclamptic twin gestations. Obstet Gynecol. (2001) 97(1):62–5. 10.1097/00006250-200104000-00035 11152909

[25] Deepashree PG Madhushankari GS Nandini DB Priya NK Ashwini R Shruthy R. Saliva as an alternative non-invasive biomarker for the estimation of uric acid levels during pregnancy: a longitudinal study. J Oral Maxillofac Pathol. (2021) 25(3):457–62. 10.4103/jomfp.jomfp_439_20 35281142 PMC8859621

[26] Koller O. The clinical significance of hemodilution during pregnancy. Obstet Gynecol Surv. (1982) 37(11):649–52. 10.1097/00006254-198211000-00001 7145246

[27] Wang X Shields CA Ekperikpe U Amaral LM Williams JM Cornelius DC. Vascular and renal mechanisms of preeclampsia. Curr Opin Physiol. (2023) 33:100655. 10.1016/j.cophys.2023.100655 37009057 PMC10062189

[28] Wang A Tian X Wu S Zuo Y Chen S Mo D Metabolic factors mediate the association between Serum uric acid to Serum creatinine ratio and cardiovascular disease. J Am Heart Assoc. (2021) 10(23):e023054. 10.1161/JAHA.121.023054 34779219 PMC9075399

[29] Santoyo JM Noguera JA Avilés F Delgado JL de Paco-Matallana C Pérez V Factors involved in endothelial dysfunction related to angiogenic disbalance and oxidative stress, in women at high risk of term Pre-eclampsia. Antioxidants. (2022) 11(7):1409. 10.3390/antiox11071409 35883900 PMC9311926

[30] Seval MM Karabulut HG Tükün A Koç AK. Cell free fetal DNA in the plasma of pregnant women with preeclampsia. Clin Exp Obstet Gynecol. (2015) 42(6):787–91. 10.12891/ceog1982.2015 26753487

[31] Bellomo G Venanzi S Saronio P Verdura C Narducci PL. Prognostic significance of serum uric acid in women with gestational hypertension. Hypertension. (2011) 58(4):704–8. 10.1161/HYPERTENSIONAHA.111.177212 21876075

[32] Nakagawa T Ana Andres-Hernando Kosugi T Sanchez-Lozada LG Stenvinkel P Kublickiene K Ananth Karumanchi S Fructose might be a clue to the origin of preeclampsia insights from nature and evolution. Hypertens Res. (2023) 46(3):646–53. 10.1038/s41440-022-01121-w 36539464 PMC10015507

[33] Borgen I Aamodt G Harsem N Haugen M Meltzer HM Brantsæter AL. Maternal sugar consumption and risk of preeclampsia in nulliparous Norwegian women. Eur J Clin Nutr. (2012) 66(8):920–5. 10.1038/ejcn.2012.61 22713766

[34] Asghar ZA Thompson A Chi M Cusumano A Scheaffer S Al-Hammadi N Maternal fructose drives placental uric acid production leading to adverse fetal outcomes. Sci Rep. (2016) 6(1):25091. 10.1038/srep25091 27125896 PMC4850405

[35] Yue C Ying C Li X. Association of first trimester serum uric acid with preeclampsia: an observational cohort study with propensity score matching. Hypertens Res. (2023) 46(2):377–85. 10.1038/s41440-022-01115-8 36539460

[36] Yakiştiran B Tanaçan A Altinboğa O Erol A Şenel S Elbayiyev S Role of derived neutrophil-to-lymphocyte ratio, uric acid-to-creatinine ratio and Delta neutrophil index for predicting neonatal outcomes in pregnancies with preeclampsia. J Obstet Gynaecol. (2022) 42(6):1835–40. 10.1080/01443615.2022.2040968 35290156

[37] Chen H Wei T Wang H Zhou Y Chen H Sun L Association of China’s two-child policy with changes in number of births and birth defects rate, 2008-2017. BMC Public Health. (2022) 22(1):434. 10.1186/s12889-022-12839-0 35246096 PMC8895506

[38] Tian ML Ma GJ Du LY Jin Y Zhang C Xiao YG The effect of 2016 Chinese second-child policy and different maternal age on pregnancy outcomes in Hebei province, China. BMC Pregnancy Childbirth. (2023) 23(1):267. 10.1186/s12884-023-05552-2 37076792 PMC10114327

[39] Li H Nawsherwan Fan C Yin S Haq IU Mubarik S Nabi G Changes in adverse pregnancy outcomes in women with advanced maternal age (AMA) after the enactment of China’s universal two-child policy. Sci Rep. (2022) 12(1):5048. 10.1038/s41598-022-08396-6 35322808 PMC8943149

[40] Murata M Fukushima K Takao T Seki H Takeda S Wake N. Oxidative stress produced by xanthine oxidase induces apoptosis in human extravillous trophoblast cells. J Reprod Dev. (2013) 59(1):7–13. 10.1262/jrd.2012-053 22986926 PMC3943235

[41] Arias-Sánchez C Pérez-Olmos A Reverte V Hernández I Cuevas S Llinás MT. Uric acid and preeclampsia: pathophysiological interactions and the emerging role of inflammasome activation. Antioxidants. (2025) 14(8):928. 10.3390/antiox14080928 40867825 PMC12382839

[42] Bainbridge SA Deng JS Roberts JM. Increased xanthine oxidase in the skin of preeclamptic women. Reprod Sci. (2009) 16(5):468–78. 10.1177/1933719108329817 19196876 PMC2992872

