# Supplementary material for: Association between the serum uric acid-to-creatinine ratio index and the risk of preeclampsia in advanced maternal age pregnant women: a retrospective cohort study
Source: Front Cardiovasc Med. 2026 Apr 2;13:1749915. doi: 10.3389/fcvm.2026.1749915 (PMC13082976; doi:10.3389/fcvm.2026.1749915)
Supplement: Supplementary file 1 [file Table1.docx]

The association between SUA/sCr index and preeclampsia(n=328) by Univariate logistic regression analysis

|  | OR | 95%CI | P-value |
| --- | --- | --- | --- |
| Age(year) | 0.98 | 0.93, 1.02 | 0.3195 |
| Parity | 0.77 | 0.64, 0.94 | 0.0106 |
| SUA/sCr | 1.15 | 1.08, 1.22 | <0.0001 |
| BMI,kg/m2 | 1.15 | 1.11, 1.19 | <0.0001 |
| Family history of hypertension |  |  |  |
| no | 1.0 |  |  |
| yes | 1.70 | 1.33, 2.17 | <0.0001 |
| ALT,U/L | 1.00 | 1.00, 1.01 | 0.0009 |
| WBC,10^9/L | 1.02 | 0.98, 1.07 | 0.3105 |
| Gravidity,num | 0.90 | 0.84, 0.96 | 0.0028 |
| RDW,% | 0.89 | 0.82, 0.97 | 0.0058 |
| RBC,10^12/L | 1.05 | 1.01, 1.10 | 0.0232 |
| Hemoglobin,g/L | 1.06 | 1.05, 1.07 | <0.0001 |
| PLT,10^9/L | 1.00 | 1.00, 1.00 | 0.0361 |
| Lymphocyte,10^9/L | 1.39 | 1.21, 1.59 | <0.0001 |
| Neutrophil,10^9/L | 1.36 | 1.31, 1.41 | <0.0001 |
| Albumin,g/L | 0.81 | 0.78, 0.84 | <0.0001 |
| ESR,mm/h | 1.05 | 1.04, 1.06 | <0.0001 |
| BUN,mmol/L | 1.16 | 1.08, 1.25 | <0.0001 |
| Potassium,mmol/L | 3.98 | 2.73, 5.80 | <0.0001 |
| Urine protein |  |  |  |
| no | 1.0 |  |  |
| yes | 2.68 | 2.10, 3.42 | <0.0001 |
