# Supplementary material for: Association between the serum uric acid-to-creatinine ratio index and the risk of preeclampsia in advanced maternal age pregnant women: a retrospective cohort study
Source: Front Cardiovasc Med. 2026 Apr 2;13:1749915. doi: 10.3389/fcvm.2026.1749915 (PMC13082976; doi:10.3389/fcvm.2026.1749915)
Supplement: Supplementary file 2 [file Table2.docx]

Supplementary Table S2

Sensitivity Analysis: Association Between SUA/sCr and Risk of Preeclampsia (Gestational Age <20 Weeks; Excluding Baseline Proteinuria; n = 1,256)

| **Exposure** | **Non-adjusted** | **Adjust I** | **Adjust II** |
| --- | --- | --- | --- |
| SUA/sCr | 1.04 (0.93, 1.16) 0.4848 | 1.05 (0.94, 1.17) 0.4240 | 1.25 (1.08, 1.45) 0.0031 |
| SUA/sCr Z-score | 1.07 (0.88, 1.30) 0.4848 | 1.08 (0.89, 1.32) 0.4240 | 1.48 (1.14, 1.93) 0.0031 |
| SUA/sCr tertile |  |  |  |
| Low | 1.0 | 1.0 | 1.0 |
| Middle | 1.36 (0.84, 2.21) 0.2141 | 1.38 (0.84, 2.25) 0.1999 | 1.00 (0.57, 1.76) 0.9968 |
| High | 1.25 (0.77, 2.03) 0.3676 | 1.25 (0.77, 2.04) 0.3707 | 1.57 (0.89, 2.76) 0.1176 |
| SUA/sCr tertile continuous | 1.11 (0.88, 1.41) 0.3825 | 1.11 (0.88, 1.41) 0.3848 | 1.28 (0.96, 1.70) 0.0963 |

Non-adjusted model adjust for: None. Adjust I model adjust for: age; gravidity; parity. Adjust II model adjust for: age ; gravidity; parity; family history of hypertension; ALT; ALB; BMI ; WBC; RBC. CI, confidence interval; OR, odds ratio
